# Neurobiology of osteoarthritis: a systematic review and activation likelihood estimation meta-analysis

**DOI:** 10.1038/s41598-023-39245-9

**Published:** 2023-08-01

**Authors:** Michelle Hall, Fiona Dobson, David Murray Klyne, Carmen Jiamin Zheng, Yuri Lopes Lima, Natalia Egorova-Brumley

**Affiliations:** 1 Centre for Health, Exercise and Sports Medicine, Department of Physiotherapy, School of Health Sciences, The University of Melbourne, Melbourne, VIC Australia; 2 Centre for Arthritis Research, School of Medicine, University College Dublin, Dublin, Ireland; 3 School of Health and Rehabilitation Sciences, University of Queensland, Brisbane, QLD Australia; 4 Melbourne School of Psychological Sciences, The University of Melbourne, Melbourne, VIC 3010 Australia; 5 School of Health Science and Social Work, Griffith University, Gold Coast, QLD Australia

**Keywords:** Cognitive neuroscience, Osteoarthritis

## Abstract

Osteoarthritis (OA) affects 240 million people worldwide. Neuroimaging has been increasingly used to investigate brain changes in OA, however, there is considerable heterogeneity in reported results. The goal of this systematic review and meta-analysis was to synthesise existing literature and identify consistent brain alterations in OA. Six databases were searched from inception up to June, 2022. Full-texts of original human studies were included if they had: (i) neuroimaging data by site of OA (e.g. hand, knee, hip); (ii) data in healthy controls (HC); (iii) > 10 participants. Activation likelihood estimation (ALE) was conducted using GingerALE software on studies that reported peak activation coordinates and sample size. Our search strategy identified 6250 articles. Twenty-eight studies fulfilled the eligibility criteria, of which 18 were included in the meta-analysis. There were no significant differences in brain structure or function between OA and healthy control contrasts. In exploratory analysis, the right insula was associated with OA vs healthy controls, with less activity, connectivity and brain volume in OA. This region was implicated in both knee and hip OA, with an additional cluster in the medial prefrontal cortex observed only in the contrast between healthy controls and the hip OA subgroup, suggesting a possible distinction between the neural correlates of OA subtypes. Despite the limitations associated with heterogeneity and poor study quality, this synthesis identified neurobiological outcomes associated with OA, providing insight for future research. PROSPERO registration number: CRD42021238735.

## Introduction

Osteoarthritis (OA) is the most common form of arthritis with an estimated 240 million people world-wide having painful OA^1^. Osteoarthritis is the most frequent reason for activity limitation in adults^1^ and can affect almost any joint, but typically affects the knees, hips, hands and feet^2^. The Osteoarthritis Research Society International definition of OA describes a complex physiology affecting multiple joint structures^3^. However, emerging evidence from anatomical and functional imaging studies of the brain^4^ is providing new insights into altered structures beyond the somatosensory correlates of the affected joint in the brain.

There is currently no cure for OA and pain is the cardinal symptom of OA. Existing non-surgical treatments (e.g. education, exercise, weight loss) have modest efficacy^5^, which are limited by a lack of understanding about how OA affects the body beyond the affected joint. As such, neuroimaging has been increasingly used to investigate brain adaptations, in the anticipation of discovering an imaging biomarker(s) that accelerates the development novel therapeutics or optimises prescription of current treatments^6^. However, interpretation of these studies is hindered due to diverse methods and experimental designs. Unsurprisingly, there is considerable heterogeneity in reported results. For example, some studies suggest that structural brain changes in OA are associated with a specific pattern of degeneration, or unique anatomical ‘brain signature’, while others report that structural changes reflect neither damage nor atrophy^7,8^. Synthesising observations across investigations is necessary to identify consistent brain alterations associated with OA to inform future research aiming to enhance OA management via a more targeted approach to treatment, in addition to the aforementioned means. Therefore, the aims of this systematic review and meta-analysis are to (1) establish the evidence for alterations in structure and function of the brain in people with OA and (2) investigate the association between changes in brain structure and function with OA joints, pain severity, and duration.

## Methods

This review was conducted in accordance with the Preferred Reporting Items for Systematic Review and Meta-analysis (PRISMA guidelines)^9^ and best practices for neuroimaging meta-analyses^10,11^. The study protocol was prospectively registered with the International Prospective Register of Systematic Reviews (PROSPERO CRD # 42,021,238,735).

### Data sources and searches

Six databases including MEDLINE via Ovid, EMBASE via Ovid, APA PsycInfo, Cumulative Index to Nursing and Allied Health Literature (CINAHL) via EBSCO, SCOPUS via Elsevier and Web of Science were searched by a librarian from inception up to 28th June, 2022. Full list of eligible outcomes was described a priori in our PROSPERO protocol (CRD42021238735). Search strategies comprised of keywords and symptoms of OA and brain measures according to the semantics of each database. The complete search strategy is presented in detail in Supplementary Appendix 1.

### Study selection

Identified studies were imported in Covidence systematic review software (Veritas Health Innovation, Melbourne Australia). Following the removal of duplicates from the initial search, two authors (NEB and MH) independently screened the articles by title and abstract to exclude irrelevant studies. Full texts of all articles considered potentially relevant by either of the two reviewers, were retrieved and screened for eligibility by both reviewers.

Studies of any design were included if they met the following criteria: (i) included people with OA diagnosed by a clinician assessment and/or field-standard criteria (e.g. American Colleague or Rheumatology, National Institute Clinical Guidelines); (ii) quantitatively report brain neuroimaging data by site of OA (e.g. hand, knee hip); (iii) brain neuroimaging data in a healthy control group; (iv) experiment included at least 10 participants in the OA group and at least 10 participants in the healthy control group, and (v) full-text human studies published as original studies in the English language.

### Data extraction

Four reviewers independently extracted data (MH, NEB, CJZ, YL) and verified data by cross-checking from all included studies. The following information was extracted: authors, publication year, type of study design, number of participants by sex, age, body mass index, disease severity, outcomes, brain regions of interest, networks, stereotactic coordinates. If multiple related contrasts were reported, we included all contrasts but handled them as one experiment, thereby using only one set of coordinates in the meta-analysis. If further information was needed, authors were contacted at least twice via email, after which data were considered irretrievable.

### Data synthesis and analysis

To perform coordinate-based meta-analysis, activation likelihood estimation (ALE) analysis was conducted using GingerALE, version 3.0.2 (https://www.brainmap.org/ale/). Studies included in the ALE analysis reported peak activation coordinates in Montreal Neurological Institute (MNI) or Talairach space and sample size. Within each experiment, the reported activation foci were treated as centres of a three-dimensional Gaussian probability distribution, whose width is determined by the study’s sample size and thus reflective of spatial uncertainty of the foci^12^. As larger samples model smaller Gaussian distributions, they are also likely to produce more reliable approximations of the “true” activation effect. Then, these modelled possibilities were combined across foci, producing a modelled activation map for each experiment. To test for spatial convergence of neuroimaging findings, voxel-wise ALE scores were calculated by taking the union of all modelled activation maps. Statistically significant convergence between experiments was identified by comparing the ALE scores against a null distribution of random spatial association, with the outcome clusters representing above-chance convergence between experiments. Correction level was set to p < 0.001, 1000 permutations and p < 0.05 cluster-level family-wise error (FWE). For illustration, the resulting ALE maps were imported to MRIcron (https://people.cas.sc.edu/rorden/mricron/install.html) and plotted over a standardised anatomical MNI-normalised template.

First, the primary contrast between OA vs healthy controls was performed. Six subsequent, exploratory analyses were performed as in previous similar ALE analysis, e.g. in fibromyalgia^13^, to contrast OA vs. healthy controls in the direction of effect as follows: (1) OA greater than healthy controls contrast (e.g. greater activation or brain volume in OA compared to healthy controls) and (2) OA less than healthy controls contrast (e.g. less activation or brain volume in OA compared to healthy controls). We also evaluated if there were imaging method-specific differences between OA and healthy controls, as follows: (3) contrast between OA vs. healthy controls as measured by resting-state functional magnetic resonance imaging; (4) contrast between OA vs. healthy controls as measured by structural MRI. Finally, we compared specific osteoarthritic joints: (5) knee OA vs. healthy controls; and (6) hip OA vs. healthy controls.

In light of the best practice guidelines for neuroimaging meta-analysis^10,11^, studies that did not report whole-brain analyses were not included. Furthermore, we conducted a pre-registered sensitivity analysis from the meta-analysis by removing studies that did not provide sufficient detail about their multiple comparison correction methods or that were not adequately corrected for multiple comparison, e.g. by reporting activation at a voxel-level threshold of p < 0.001 (uncorrected) with an additional cluster-level correction of p < 0.05.

### Study quality and risk of bias

Methodological quality of the studies was assessed independently by two reviewers (FD and DMK) using the 14-item National Institute of Health Quality Assessment Tool for Observational Cohort and Cross-Sectional Studies^14^. Consensus on items was achieved by a staged learning approach where the reviewers met to check understanding initially after independently rating three studies, then again after completing all ratings. All conflicted items were then resolved by a final consensus. Overall study quality was assessed based on the potential risk of bias across four domains; (1) information bias (item 1), (2) selection bias (items 2, 3, 4), (3) measurement bias (items 5, 6, 7, 8, 9, 10, 11, 12, 13), and (4) confounding bias (item 14), according to the tool guidelines^14^. A domain was considered to be a “potential risk of bias” if at least one item within the domain received a “no” response, or most items in the domain received a “cannot determine/not reported” response. As the analyses included in this review were cross-sectional, items 6, 7, 10 and 13 within the measurement bias domain were either rated as “no” or “not applicable” for all studies. To account for this issue, we omitted these items for the scoring of domain bias and enforced a maximum study quality score of “moderate” (rather than “high”), i.e. when no domains were considered a potential source of bias. Study quality was downgraded to “low” or “very low” if one or more domains, respectively, were considered a potential source of bias.

### Conference presentation

The results reported in this manuscript have been previously presented at the Australian Brain and Psychological Sciences Meeting, with the abstract published in the Conference Booklet. www.abps2022.org/wp-content/uploads/2022/07/ABPS2022-conference-booklet.pdf.

## Results

**Figure 1:**
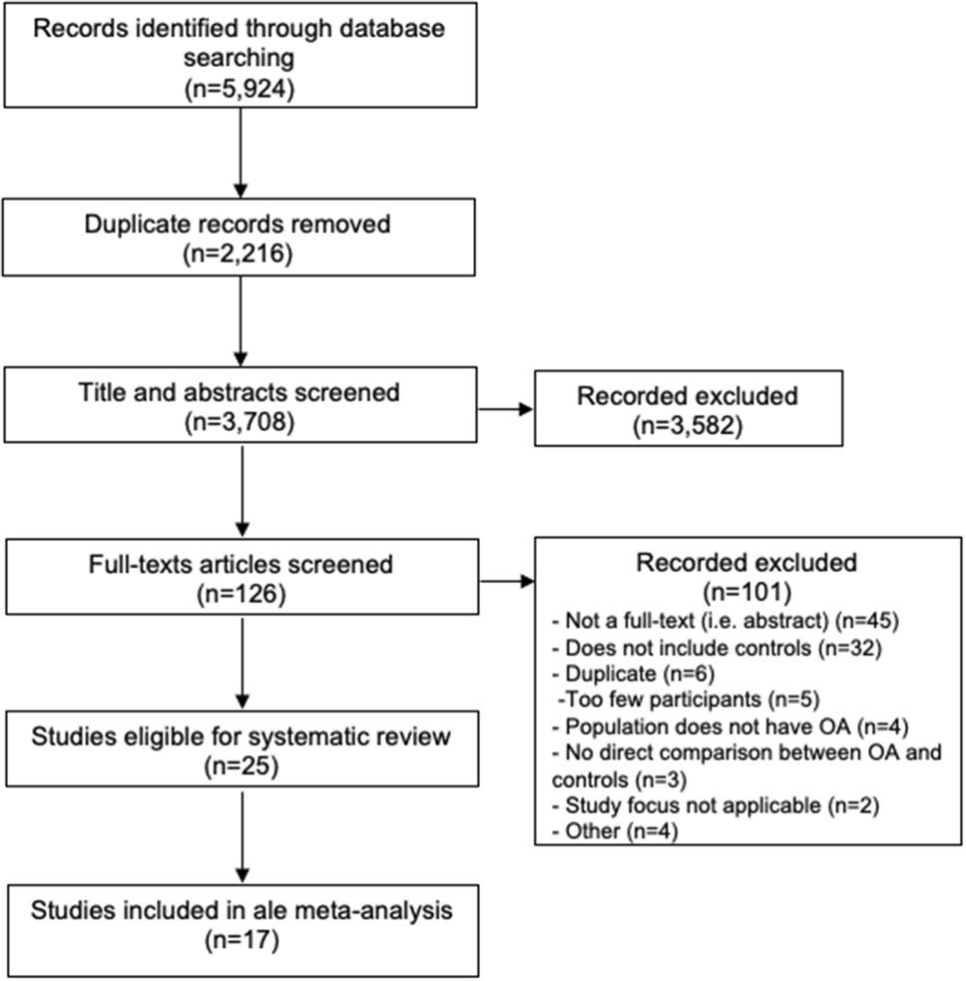
PRISMA flowchart of the study selection process.

Our search strategy identified 6250 articles (Fig. 1). Twenty-eight studies fulfilled the eligibility criteria studies and 18 of these studies were included in meta-analysis. Characteristics of studies are described in Table 1. The majority (n = 19) of experiments^7,15–32^ evaluated knee OA, four evaluated hip OA^8,33–35^, three evaluated hand OA^36–38^, and two evaluated hip and knee OA^39,40^. A summary of the imaging outcomes is described in Table 2.

**Table 1:** Summary characteristics of included studies.

Author (year)	Study design	Joint(s) affected	Inclusion criteria for OA	Group, number, (% females)	Age yrs, mean (SD)	Body mass index kg/m^2^, mean (SD)	Radiographic disease severity	Pain duration mean (SD)	Pain intensity mean (SD)
Alshuft et al. (2016)	Cross-sectional	Knee	Radiological OA with pain lasting for ≥ 3 months and experienced most of the day on most days of the week in the last month	OA: 40 (53%) Control: 30 (57%)	OA: 66.1 (8.5) Control: 62.7 (7.4)	OA: 28.8 (4.9) Controls: 26.2 (4.9)	NR	102.1 (NR) months	VAS^1^ = 3.2
Baliki et al. (2011)	Cross-sectional	Knee	Clinician-based diagnosis of OA	OA: 30 (20%) Control: 46 (30%)	OA: 53.5 (7.5) Control: 38.8 (12.5)	NR	NR	12.2 (9.5) years	VAS^1^ = 5.8 (1.8)
Baliki et al. (2014)	Cross-sectional	Knee	Clinician-based diagnosis of OA	OA: 14 (43%) Control: 36 (67%)	OA: 58.3 (9.9) Control: 41.4 (12.3)	NR	NR	11.0 (9.2) years	VAS^1^ = 6.1 (2.1)
Barroso et al. (2020)	Cross-sectional	Knee and hip	OA diagnosis according to ACR criteria with indication for total joint replacement	Knee OA: 91 (79%) Hip OA: 24 (33%) Control: 36 (56%)	Knee OA: 65.5 (6.5) Hip OA: 59.7 (8.2) Control: 59.2 (8.0)	Knee OA: 30.4 (4.9) Hip OA: 28.3 (3.7) Control: 27.8 (4.6)	Knee OA: KL1 = 1.8% KL2 = 23.1% KL3 = 39.1% KL4 = 22.8% Hip OA: KL1 = 0% KL2 = 0% KL3 = 25% KL4 = 75%	Knee OA: 7.7 (6) years Hip OA: 5.1 (4.3) years	Knee OA: NRS^1^ = 6.6 (1.7) Hip OA: NRS^1^ = 6 (1.6)
Barroso et al. (2021)	Cross-sectional	Knee and hip	OA diagnosis according to ACR criteria with indication for total joint replacement	Knee OA discovery: 46 (65%) Knee OA testing: 45 (84%) Hip OA testing: 23 (40%) Control: 35 (57.1%)	Knee OA discovery: 65.3 (7.4) Knee OA testing: 65.8 (5.6) Hip OA testing: 59.5 (7.4) Control: 59.5 (7.9)	Knee OA discovery: 30.0 (4.4) Knee OA testing: 30.9 (5.5) Hip OA testing: 28.8 (3.4) Control: 28.2 (4.6)	Knee OA discovery: KL1 = 2.2% KL2 = 26.1% KL3 = 45.7% KL4 = 26.1% Knee OA testing: KL1 = 0% KL2 = 20.0% KL3 = 48.9% KL4 = 31.1% Hip OA testing = KL1 = 0% KL2 = 0% KL3 = 21.7% KL4 = 72.2%	Knee OA discovery: 6.8 (5.5) years Knee OA testing: 8.5 (6.4) years Hip OA testing = 5.03 (4.2) years	Knee OA discovery: NRS^1^ = 6.5 (1.4) Knee OA testing: NRS^1^ = 6.8 (1.9) Hip OA testing: NRS^1^ = 6.2 (1.6)
Cheng et al. (2022)	Cross-sectional	Knee	OA diagnosis according to ACR	OA: 166 (75%) Control: 88 (64%)	OA: 52.9 (5.2) Control: 53.8 (4.8)	OA: 24.0 (2.9) Control: 24.0 (2.8)	NR	46.0 (50.2) months	VAS^1^ = 4.3 (1.3)
Cottam et al. (2016)	Cross-sectional	Knee	Radiographic diagnosis of OA	OA: 26 (54%) Control: 27 (67%)	OA: (median) 67.5 (7.5) Control: (median) 65 (5.8)	NR	NR	NR	VAS^2^ = 40.2 (18)
Cottam et al. (2018)	Cross-sectional	Knee	Radiographic diagnosis of OA	OA: 25 (52%) Control: 19 (58%)	OA: (median) 65 (8.0) Control: (median) 65 (7.3)	NR	NR	NR	VAS^2^ = 27.8 (17.5)
El-Najjar et al. (2020)	Cross-sectional	Knee	OA diagnosis according to ACR	OA: 45 (73%) Control: 15 (60%)	OA: 57.0 (6.0) Control: 59.5 (9.2)	NR	NR	5.7 (2.4) years	VAS^2^ = 72 (16)
Gandola et al. (2017)	Cross-sectional	Trapeziometacarpal joint	Diagnosis of rhizartrosis, indication to surgery	OA: 35 (77%) Controls: 35 (77%)	OA: 60.1 (9.4) Control: 57.9 (9.9)	NR	Eaton-Litter I = 6% II = 29% III = 26% IV = 6%	40.7 (NR) months	VAS^1^ = 4.3 (2.9)
Gwilym et al. (2010)	Pre-post design	Hip	Diagnosis of primary OA with unilateral right-sided hip pain with indication for total hip arthroplasty	OA: 16 (50%) Control: 16 (50%)	68 (NR)	OA = 27.0 (1.5) Controls = 24.5 (1.0)	NR	NR	VAS^1^ (median) = 5
Hiramatsu et al. (2014)	Cross-sectional	Knee	Primary and secondary OA on the right side, pain > 3 months, pain ≥ 3/10 on NRS	OA: 12 (75%) Control: 11 (73%)	OA: 62.7 (5.7) Control: 56.4 (7.3)	NR	KL1 = 17% KL2 = 58% KL3 = 25%	113.4 (171.6) months	NRS^1^ = 5.3 (2.3)
Howard et al. (2012)	Cross-sectional	Carpometacarpal	Diagnosis of OA according to ACR, resting pain ≥ 3 during last week on NRS	OA: 16 (100%) Control: 17 (100%)	OA: 60.9 (NR) Control: 64.2 (NR)	NR	NR	NR	NRS^1^ = 3.7 (NR)
Iwabuchi et al. (2020)	Cross-sectional	Knee	Self-reported diagnosis osteoarthritis and/or chronic knee pain	OA: 44 (50%) Control: 29 (38%)	OA: 62.8 (8.6) Control: 64.4 (11.1)	NR	NR	119.7 (121.9) months	NRS^2^ = 36.3 (29.4)
Kang et al. (2022)	Cross-sectional	Knee	Radiographical diagnosis of OA with pain that could not be relieved with non-surgical treatment	OA: 37 (92%) Control: 37 (81%)	OA: 71.6 (5.6) Control: 69.5 (5.4)	NR	All KL3 or KL4	NR	NR
Lan et al. (2020)	Cross-sectional	Knee	Age ≥ 65y with diagnosis of OA made from medical history and imaging	OA: 23 (65%) Control: 23 (61%)	OA: 71.2 (4.2) Control: 71.4 (4.1)	NR	NR	NR	NRS^1^ = 3.2 (1.9)
Lewis et al. (2018)	Cross-sectional	Knee	Pain ≥ 3/10 on ≥ 3 days per week in past month and awaiting TKA	OA: 29 (48%) Control: 18 (61%)	OA: 68.0 (10.0) Control: 71.0 (8.0)	OA: 31.0 (5.7) Control: 24.9 (3.0)	NR	NR	NRS^1^ = 5.2 (2.3)
Liao et al. (2018)	Cross-sectional	Knee	Diagnosis of OA according to ACR	OA: 30 (87%) Control: 30 (87%)	OA: 56.5 (6.8) Control: 55.2 (5.7)	NR	NR	7.3 (5.1) years	VAS^1^ = 5.1 (1.8)
Mao et al. (2016)	Cross-sectional	Knee	Diagnosis of OA according to ACR and no history of other pain conditions, pain ≥ 3/10 for > 6 months	OA n = 26 (85%) Control: 31 (84%)	OA: 55.5 (9.1) Control: 53.1 (6.4)	NR	NR	7.3 (9.3) years	VAS^1^ = 4.5 (1.8)
Mutso et al. (2012)	Cross-sectional	Knee	Diagnosis not described	OA: 20 (20%) Control: 50 (56%)	OA: 53.1 (7.5) Control: 40.1 (11.3)	NR	NR	NR	NR
Railton et al. (2022)	Cross-sectional	Hip	Hip OA requiring total hip replacement	OA: 30 (65%) Control: 10 (60%)	OA: 56.0 (9.0) Control: 52.9 (6.5)	OA: 28.0 (4.3) Control: 25.0 (4.6)	NR	> 1 year	NR
Reckziegel et al. (2016)	Cross-sectional	Knee	Radiographical diagnosis of OA and pain during most of the day for most days the past month	OA: 14 (36%) Control: 14 (64%)	OA: 64.1 (7.4) Control: 62.0 (6.6)	NR	NR	7.7 (4.9) years	VAS^2^ = 29.0 (28.4)
Rodriguez-Raecke et al. (2013)	Cross-sectional	Hip	Unilateral primary hip OA scheduled for total hip replacement	OA: 20 (50%) Control: 20 (50%)	OA: 63.3 (9.5) Control: 61.0 (8.5)	NR	NR	7.4 (8.0) years	VAS^2^ = 65.5 (12.5)
Rodriguez-Raecke et al. (2009)	Cross-sectional and longitudinal	Hip	Unilateral primary hip OA scheduled for total hip replacement	OA: 32 (59%) Control: 32 (60%)	OA: 66.8 (9.0) Control: 63.9 (8.8)	NR	NR	7.4 years	NR
Russell et al. (2018)	Pre-post design	Hand	Age 40-75y, diagnosis of OA according to ACR, pain ≥ 5 on NRS	OA: (86%) Control: 11 (82%)	OA: 62 (7.7) Control: 59 (7.4)	NR	NR	NR	NR
Tétreault et al. (2018)	Cross-sectional	Knee	Diagnosis of OA according to ACR	OA: 39 (56%) Control: 20 (50%)	OA: 58.7 (7.6) Control: 57.9 (6.7)	NR	NR	NR	VAS^1^ = 6.2 (NR)
Ushio et al. (2020)	Cross-sectional	Knee	OA diagnosis, VAS > 30/100, pain duration > 3 months	OA: 19 (100%) Control: 15 (100%)	OA: 73.2 (5.1) Control: 74.9 (4.6)	NR	KL3 = 32% KL4 = 68%	102.9 (88.6) months	VAS^2^ = 64.5 (15.1)
Weerasekera et al. (2021)	Pre-post design	Knee	Age 40–85, diagnosis of OA schedule to primary unilateral TKA	OA: 34 (53%) Control: 13 (46%)	OA: 66.1 (8.2) Control: 49.4 (17.0)	NR	NR	NR	WOMAC pain^3^ = 9.4 (3.9)

*OA* osteoarthritis, *NR* not reported, *KL* Kellgren Lawrence radiographic disease grade, *ACR* American College of Rheumatology, *TKR* total knee arthroplasty, *NRS* numeric rating scale, *WOMAC* Western Ontario and McMaster Universities Arthritis Index, *VAS* visual analogue scale.

^1^range: 0–10, ^2^range 0–100, ^3^range 0–20.

**Table 2:** Summary of imaging outcomes.

Author	Imaging method	Brain measure	Direction of effect	Whole brain/regions/networks	Results of brain regions analysis	Results of brain networks analysis	Correction level	Coordinate system/seed (yes/no/na)	Included in ALE (yes/no)
Alshuft et al. (2016)	MRI structural	Cortical thickness	OA < control	Whole brain	R anterior insula	NA	Uncorrected < 0.001	Tal (NA)	No
Baliki et al. (2011)	MRI structural	Gray matter volume; Gray matter density	OA < control	Whole brain	B S2/posterior insula R anterior insula B hippocampus R paracentral lobule L M cingulum M occipital	NA	p = 0.05	MNI (yes)	Yes
Baliki et al. (2014)	MRI functional resting state	Connectivity	OA < control	DMN; Salience network; Sensorimotor network; R frontoparietal network; Visual networks	M PFC ACC L anterior insula/IFG L SMG	DMN	FWE cluster corrected p < 0.01	MNI (yes)	Yes
Barroso et al. (2020)	MRI structural	Gray matter volume; Regional gray matter density; Gray matter volume in ROIs	(Knee & Hip) OA < Control (flipped)	Whole brain	L precentral gyrus R temporal lobe R anterior cingulate gyrus	NA	p < 0.001, minimum cluster k = 66	MNI (yes)	Yes
			Knee OA < Control (flipped)		R precuneus cortex				
			Knee OA > Control (flipped)		L MFG				
Barroso et al. (2021)	MRI functional resting state	Connectivity	OA < control	Whole brain	L paracingulate cortex R lateral occipital cortex R postcentral gyrus R insula	DMN Cingulo-opercular Auditory SN Frontoparietal cortex	FDR correction for multiple comparisons, α = .05	MNI (yes)	Yes
			OA > control		R precentral gyrus L postcentral gyrus L temporofusiform gyrus R precentral gyrus L postcentral gyrus R temporal fusiform gyrus				
Cheng et al. (2022)	MRI structural	White matter	OA < control	Whole brain	OA > control in FA values body of corpus callosum, splenium of corpus callosum, bilateral superior longitudinal fasciculus, cingulum, bilateral superior corona radiata, R posterior corona radiata	NA	p < 0.05 and corrected by the threshold-free cluster enhancement (TFCE)method	NA	No
			OA > control		OA < control in MD, AD, and RD values the genu of corpus callosum, body of corpus callosum, splenium of corpus callosum, corona radiata, R posterior thalamic radiation, superior longitudinal fasciculus, middle cerebellar peduncle				
Cottam et al. (2016)	MRI ASL	Regional CBF	Non-significant	Whole brain	No significant difference in global or regional CBF	NA	FWE correction *p* < 0.05	NA	No
Cottam et al. (2018)	MRI functional resting state	Connectivity	OA > control	Whole-brain; SN, CEN, DMN	R anterior insula*, L lingual gyrus R anterior insula*, L precuneus R anterior insula*, L MFG R anterior insula*, L posterior cingulate R anterior insula*, R lateral occipital gyrus R anterior insula*, L angular gyrus	DMN	FWE correction *p* < 0.05 at cluster level	MNI (yes)	Yes
			OA < control		L DLPFC*, R temporal pole				
El-Najjar et al. (2020)	MRI MRS	Myo-inositol:Glx	OA > control	Regional	M ACC	NA	Uncorrected < 0.05	NA	No
Gandola et al. (2017)	MRI functional task	BOLD signal	OA < control	Whole-brain	L precentral gyrus L postcentral gyrus R primary motor cortex	NA	FWE correction p < 0.05 at voxel level	MNI (yes)	Yes
Gwilym et al. (2010)	MRI structural	Gray matter volume	OA < control	Whole-brain	B medial thalamus	NA	Uncorrected p < 0.001	MNI (yes)	Yes
			OA > control		L anterior insula L amygdala B temporal fusiform cortex cerebellum R posterior parahippocampal gyrus L OFC B occipital cortex				
Hiramatsu et al. (2014)	MRI functional task	BOLD signal	OA > control	Whole-brain	B superior frontal cortex L inferior parietal cortex R lingual gyrus L superior occipital cortex L middle occipital cortex	NA	Uncorrected p < 0.001 at voxel level	MNI (yes)	Yes
Howard et al. (2012)	MRI ASL	Regional CBF	OA > control	Whole-brain	B medial frontal gyrus L MFG L IFG L precentral gyrus R precentral gyrus B precuneus L superior parietal lobule B inferior parietal lobule L superior temporal gyrus L middle temporal gyrus L inferior temporal gyrus L fusiform gyrus B cuneus L lingual gyrus L middle occipital gyrus L inferior occipital gyrus	NA	corrected for multiple comparisons p < 0.05 at cluster level	MNI (yes)	Yes
Iwabuchi et al. (2020)	MRI ASL	Regional CBF	OA > control	Whole-brain	L lateral occipital cortex B cerebellum L fusiform gyrus R inferior temporal gyrus B lingual gyrus L brain stem L temporal pole R thalamus B parahippocampal gyrus L frontal pole L caudate	DMN SN	Uncorrected for multiple comparisons p < 0.05	MNI (yes)	Yes
			OA < control		L SMG R frontal pole R cerebellum L Heschl’s gyrus L ACC M OFC R anterior insula R opercular cortex L cerebellum R postcentral gyrus L frontal pole midcingulate gyrus B OFC R precentral gyrus B lateral occipital cortex R MFG R ACC R IFG L inferior temporal gyrus L superior temporal gyrus R SFG L planum temporale L frontal pole L angular gyrus				
Kang et al. (2022)	MRI structural	Gray matter volume	OA < control	Whole-brain	L middle temporal gyrus L inferior temporal Gyrus	NA	AlphaSim corrected p < 0.05 combined with uncorrected	MNI (no)	Yes
	MRI functional resting state	Connectivity	OA < control	L MTG*, Whole-brain	L MTG*, R dorsolateral SFG, L MTG*, L MFG, L MTG*, L medial SFG		voxel-wise p < 0.001		
Lan et al. (2020)	MRI functional resting state	ALFF; connectivity	OA < control	Whole-brain	B precuneus gyrus B angular gyrus L medial SFG	DMN	voxel-wise p < 0.001, cluster wise p < 0.025 for each tail	MNI (yes)	Yes
			OA > control		B cerebellum B amygdala L precuneus gyrus*, R supplementary motor area				
Lewis et al. (2018)	MRI structural	White matter structure (FA); grey matter density	Grey matter density: OA < control	Whole-brain	Ipsilateral S1 Contralateral NAc Ipsilateral Nac Contralateral amygdala Ipsilateral amygdala	NA	corrected for multiple comparisons p < 0.05 using threshold-free cluster enhancement	MNI (yes)	Yes
			FA: OA < control		Midbrain				
Liao et al. (2018)	MRI structural	Gray matter volume	OA < control	Whole-brain	B OFC R lateral PFC R precentral and postcentral cortex	NA	FWE corrected p < 0.05	MNI (yes)	Yes
Mao et al. (2016)	MRI structural	Gray matter volume	OA < control	Subcortical structures	B caudate nucleus	NA	Multiple comparisons corrected p < 0.025	NA	No
Mutso et al. (2012)	MRI structural	Gray matter volume	NS	Hippocampus	–	NA	NA	MNI (no)	No
Railton et al. (2022)	MRI functional resting state	Connectivity	OA < control	S2*, anterior/posterior insulae*, thalamus*, Whole-brain	OA < control Lateral posterior insula, motor cortices	NA	p < 0.05, FDR threshold of 0.05, corresponding to a cluster volume of greater than 322 voxels, as determined by AlphaSim	NA	No
			OA > control		S2 L posterior insula				
Reckziegel et al. (2016	MRI MRS	GABA level	non-significant	Regional	M ACC	Salience network	Uncorrected a priori p < 0.05	NA	No
Rodriguez-Raecke et al. (2013)	MRI structural	Gray matter density	OA < control	Whole-brain	OA < control L ACC R insula R cerebellum R pars orbitalis L SFG L middle temporal gyrus R superior medial gyrus R pars opercularis R DLPFC R superior temporal gyrus	NS	Uncorrected p < 0.001	MNI (yes)	Yes
			OA > control		R putamen				
Rodriguez-Raecke et al. (2009)	MRI structural	Gray matter density	OA < control	Whole-brain	B ACC R amygdala R DLPFC L midcingulate cortex B insular cortex R brainstem L medial temporal gyrus B midorbital gyrus R SFG R medial temporal pole R cerebellum R superior medial gyrus R S1	NA	Uncorrected p < 0.001	MNI (yes)	Yes
Russell et al. (2018)	MRI structural	Gray matter volume	OA < control	a priori ROIs	ACC	NA	FWE correction p = 0.05	MNI (yes)	No
Tétreault et al. (2018)	MRI structural MRI functional resting state	Gray matter density; connectivity	Degree count: OA < control	Whole-brain	L frontal pole R paracingulate gyrus L posterior cingulate gyrus R insula R parietal operculum cortex	NA	5000 random permutations followed by threshold free cluster enhancement correction, which accounts for multiple comparison	MNI (yes)	Yes
			OA > control		L ACC L postcentral gyrus L thalamus				
			Grey matter density: OA < control		L frontal pole L middle temporal gyrus R central opercular cortex				
			OA > control		R PAG R caudate				
Ushio et al. (2020)	MRI functional resting state	Connectivity	OA > control	Anterior insula*, Whole-brain	L anterior insula*, R OFC R anterior insula*, R OFC R anterior insula*, B frontal pole	NA	p < 0.001 for the uncorrected peak-level, p < 0.05 FWE correction at cluster level	MNI (coordinates only for the regions, no seeds available)	Yes
Weerasekera et al. (2021)	MRI MRS	Myoinosital	OA > control	L thalamus	L thalamus	NA	p = 0.05	MNI (NA)	No
		NAA	OA < control	L thalamus	L thalamus	NA	p = 0.05	MNI (NA)	

*NA* not assessed; *R* right side; *L* left side; *B* bilateral; *M* medial; *MNI* Montreal Neurological Institute; *Tala* Talairach; *Flip* to examine brain hemisphere contralateral to pain site; *ACC* anterior cingulate cortex; *ALFF* amplitude of low frequency fluctuation; *BOLD* blood-oxygen level dependent signal; CBF cerebrospinal fluid; *CEN* central executive network; *DLPFC* dorsolateral prefrontal cortex; *DMN* default mode network; *FA* fractional anisotropy; *FDR* false discovery rate; *FWE* family wise error; *IFG* inferior frontal gyrus; *MFG* middle frontal gyrus; *MRS* magnetic resonance spectroscopy; *Myo*-*inositol*:*Glx* ratio between myoinositol and glutamate plus glutamine (Glx) as measurement of neurometabolite; *NaA* N-acetylaspartate; *NAc* nucleus accumbens; *OFC* orbitofrontal cortex; *PAG* periaqueductal gray; *PFC* prefrontal cortex; *S1* primary somatosensory cortex; *S2* secondary somatosensory cortex; *SFG* superior frontal gyrus; *SMG* supramarginal gyrus; *SN* salience network.

*Seed regions in connectively analyses.

### Primary coordinate-based (ALE) meta-analysis

**Figure 2:**
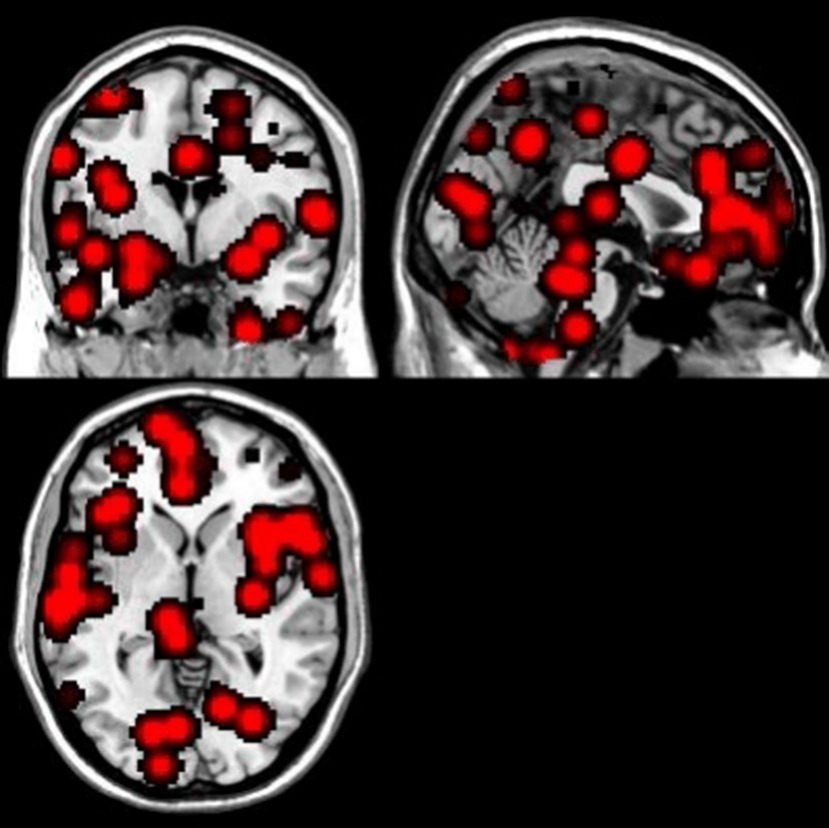
Distribution of foci from all experiments reporting differences between people with osteoarthritis and healthy controls. No significant clusters were identified in the osteoarthritis vs. healthy control contrast in the ALE analysis.

Our primary pre-registered meta-analysis evaluated whether differences between people with OA and healthy controls existed, regardless of the sign of the association. No differences were observed (Fig. 2) based on data from 18 experiments^7,8,16,19,21,22,24–26,30,31,33–37,39,40^, including 1102 participants. Eleven experiments included knee OA only^7,16,19,21–26,30,31^, three included hip OA only^8,33,35^, two included hip and knee OA^39,40^, and two included hand OA only^36,37^. Imaging methods included MRI structural (n = 9, Ref.^7,8,23,25,26,30,33,35,39^), fMRI resting state (n = 7, Ref.^16,19,23,24,30,31,40^), fMRI task (n = 2, Ref.^21,36^), and MRI arterial spin labelling (n = 2, Ref.^22,37^). Twelve experiments excluded participants based on the presence of psychiatric co-morbidities, such as depression^7,16,21,22,24–26,31,33,34,39,40^, albeit to varying severities. Six experiments did not provide eligibility criteria related to psychiatric comorbidities^8,19,30,35–37^. Our sensitivity analysis that included only experiments with appropriate correction (n = 11, including n = 667 participants), implicated the left post central gyrus in OA.

### Exploratory coordinate-based (ALE) meta-analysis

**Figure 3:**
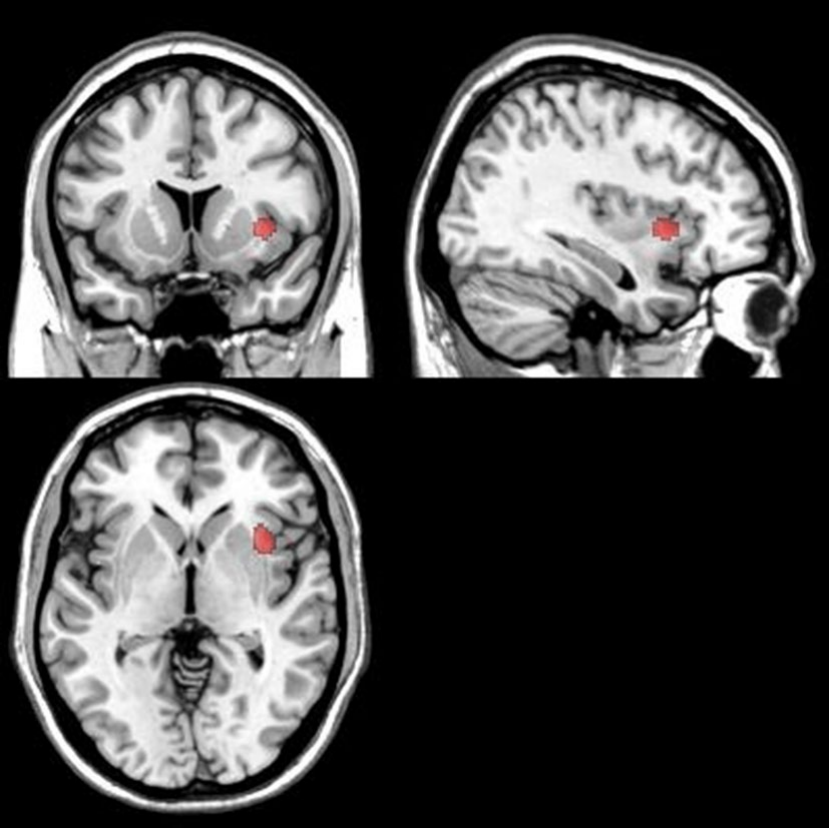
Results for the osteoarthritis vs. healthy control contrast by effect direction. A significant cluster in the right insula was observed for the osteoarthritis < healthy control contrast. No significant results were observed for the osteoarthritis > healthy control contrast.

Ten experiments reported greater activation, connectivity or brain volume in OA than in healthy controls^8,19,21,22,24,30,31,33,37,40^, including 534 participants, and 16 experiments reported less activation, connectivity or brain volume in OA than in healthy controls^7,8,16,19,22–26,30,33,35,36,39,40^, including 1163 participants. Our meta-analysis found no significant differences for the dataset that reported OA greater than healthy controls results. In contrast, we observed a significant cluster in the right anterior insula (Fig. 3) associated with data showing OA less than healthy controls. Ten experiments on 730 participants used structural MRI techniques and seven experiments on 457 participants used functional MRI studies (Table 1). No significant results were observed when OA vs. healthy control groups were compared separately for the selection of studies using homogenous imaging methods.

**Figure 4:**
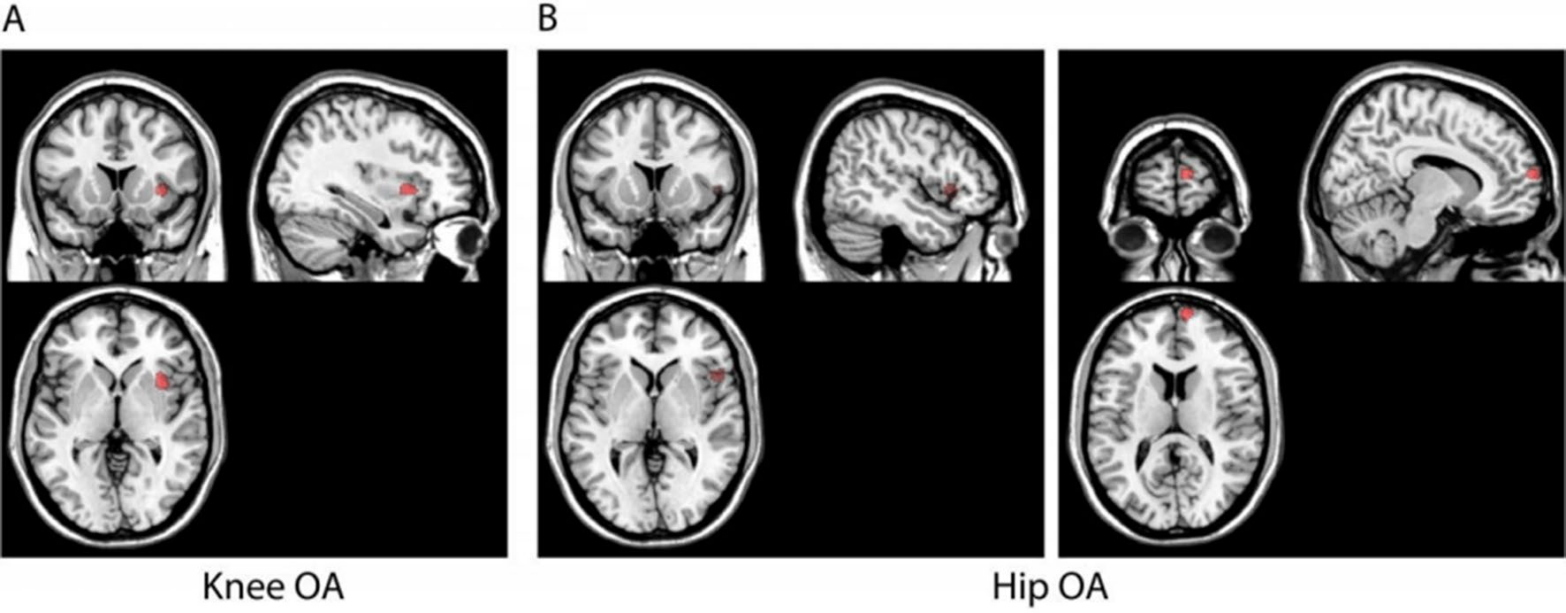
Results for the differences between osteoarthritis and healthy controls by osteoarthritis site. (**A**) shows a significant cluster in the right insula in knee osteoarthritis vs. healthy controls. (**B**) shows 2 significant clusters, in the right insula and the medial prefrontal cortex in hip osteoarthritis vs. healthy controls. No significant clusters were observed for hand osteoarthritis vs. healthy controls (not shown).

Thirteen experiments^7,16,19,21–26,30,31,39,40^ comparing knee OA to healthy controls, included a total of 863 participants revealed a significant cluster in the right insula (Fig. 4A). Three experiments^8,33,35^ comparing hip OA to healthy controls included 136 participants and revealed two clusters in the right insula and the medial prefrontal cortex (Fig. 4B). Only two experiments compared hand OA to healthy controls, and due to limited data available a meta-analysis was not performed. Coordinates for each a priori but unregistered exploratory contrasts are provided in Supplementary Appendix 2.

### Narrative synthesis of contrasts between OA and healthy controls

Ten experiments were not eligible for inclusion in the meta-analysis. Nine experiments compared knee OA to healthy controls^15,17,18,20,23,27,28,32,38^, and one compared hand OA to healthy controls^38^. Five used structural MRI^15,17,27,28,38^, three used MRS MRI^20,29,32^, one used functional MRI^34^ and one used atrial spin labelling MRI^18^. There are two reports of lower gray volume matter in hand OA^38^ and knee OA^27^, and another report of no significant differences in knee OA^28^. Studies reported no differences between knee OA and healthy control groups for regional cerebrospinal fluid^18^, gamma-aminobutyric acid (GABA) level^29^, metabolites including myoinosital or N-acetyl aspartate^32^.

Across all eligible studies, the most consistently implicated brain regions in OA were the following: the insula (12 experiments)^7,8,15,16,19,22,30,31,33–35,40^; medial frontal regions, including orbito-frontal, middle (pre)frontal gyrus and superior frontal areas (10 experiments); paracentral regions, including pre and post-central regions, S1/S2 (14 studies); cingulate, including anterior and mid portions (10 experiments)^8,16,19,20,22,30,35,38–40^, precuneus (4 experiments)^19,22,24,40^; amygdala (4 experiments)^24,25,33,35^; and parahippocampal area, including the lingual gyrus (4 experiments)^19,21,22,37^/hippocampus (3 experiments)^7,22,33^ and fusiform regions (5 experiments)^21,22,33,37,40^. While our meta-analysis focused on whole-brain studies, several studies exploring the neurobiology of OA focused on network changes. Five studies reported differences in the default-mode network (DMN)^16,19,22,24,40^, and three studies in the salience network^22,29,40^.

### Summary of the association between brain measures and pain

Fifteen studies assessed correlations with pain intensity (Table 3). Four studies showed an association between insula connectivity/nodal degree and increased pain intensity^16,19,31,40^ and two studies reported that higher GABA levels were associated with increased pain intensity^20,29^. Eight studies assessed the correlation with pain duration^7,15–17,20,26,39^, with four correlations reaching statistical significance (Table 3).

**Table 3:** Correlations between brain imaging measures and pain duration and pain intensity.

	Osteoarthritic joint	Pain duration	Correlation value	P value	Pain intensity	Correlation r value, unless otherwise stated	P VALUE
Alshuft et al. (2016)	Knee	**Cortical –thickness of total brain—months**	**-0.46**	**0.01**	NR	NR	NR
Baliki et al. (2011)	Knee	**Gray matter reorganisation – years** Gray matter density – years	**0.61** NR	** < 0.01** NS (NR)	Gray reorganisation – VAS Gray matter density – VAS	NR NR	NS (NR) NS (NR)
Baliki et al. (2014)	Knee	**High frequency power within the default mode network – years**	**0.77**	** < 0.01**	High frequency power within the default mode network – VAS	– 0.19	NS (NR)
		Phase differences between the default mode network and frontoparietal network	0.64	0.53	Phase differences between the default mode network and frontoparietal network – VAS	0.13	NS (NR)
		Size of the default mode network	-0.10	NS (NR)	Size of default mode network -VAS	– 0.01	NS (NR)
					**Medial prefrontal cortex – insular** **connectivity—VAS**	**0.61**	** < 0.05**
Barroso et al. (2021)	Knee & Hip	Nodal topology—years	NR	NS (NR)	**Nodal topology (Multinodal distributed degree properties – increased degree (i.e. inferior temporal gyrus; paracingulate cortex; insula; lateral occipital cortex) and decreased degree (i.e. putamen; operculum; middle frontal gyrus; parahippocampus)—VAS**	**0.84**	**p < 0.001**
Barroso et al. (2020)	Hip	Non-flipped brain analysis Paracingulate gyrus, cingulate gyrus, anterior division, juxtaposicioNRl lobule cortex – years	0.20	NS (NR)	Non-flipped brain analysis Paracingulate gyrus, cingulate gyrus, anterior division, juxtaposicioNRl lobule cortex—NRS	– 0.16	NS (NR)
	Hip	Flipped brain analysis Cingulate gyrus, anterior division, posterior division – years	-0.16	NS (NR)	Flipped brain analysis Cingulate gyrus, anterior division, posterior division – NRS	– 0.27	NS (NR)
	Hip & Knee	Precentral gyrus – years	0.03	NS (NR)	Precentral gyrus – NRS	0.10	NS (NR)
	Hip & Knee	Temporal pole – years	0.08	NS (NR)	Temporal pole – NRS	0.03	NS (NR)
	Knee	Precuneous cortex, intracalcarine cortex – years	-0.06	NS (NR)	Precuneous cortex, intracalcarine cortex – NRS	0.04	NS (NR)
	Knee	Middle frontal gyrus, superior frontal gyrus – years	0.12	NS (NR)	Middle frontal gyrus, superior frontal gyrus – NRS	0.01	NS (NR)
Cheng et al. (2022)	Knee	White matter—years	NR	> 0.05	White matter—VAS	NR	> 0.05
Cottam et al. (2016)	Knee	NR	NR	NR	Amygdala – cerebral blood flow	0.50	NR
					Hippocampus – cerebral blood flow	0.57	NR
					Anterior mid-cingulate cortex – cerebral blood flow	0.61	NR
Cottam et al. (2018)	Knee	NR	NR	NR	**Right anterior insula functional connectively with: Posterior cingulate cortex**	**t (22) = 2.68**	**0.015**
					**Superior frontal gyrus**	**t (22) = 2.1**	**0.048**
El-NRjjar et al. (2020)	Knee	**Myo-inositol: gluatamate and gluatamine – years**	**0.61**	**0.0001**	**Mid-anterior cingulate cortex gamma-aminobutyric acid – VAS**	– **0.86**	** < 0.001**
					Glutamate and glutamine – VAS	0.09	0.55
					**Myo-inositol: gluatamate and gluatamine – VAS**	**0.40**	**0.02**
Gwilym et al. (2010)	Hip	NR	NR	NR	Cerebellum gray matter volume—VAS	NR	NR
Hiramatsu et al. (2014)	Knee	NR	NR	NR	NR	NR	NR
Iwabuchi et al. (2020)	Knee	NR	NR	NR	Cerebral blood flow – NRS	NR	NS (NR)
Lewis et al. (2018)	Knee	NR	NR	NR	Contralateral amygdala—NRS	0.30	0.13
					Ipsilateral amygdala—NRS	0.18	0.35
					Contralateral nucleus accumbens—NRS	0.03	0.88
					Ipsilateral nucleus accumbens—NRS	0.01	0.95
					Ipsilateral primary somatosensory cortex—NRS	0.24	0.22
					Fractional anisotropy—NRS	– 0.12	0.54
Liao et al. (2018)	Knee	Gray matter (volume)—years	– 0.144	0.448	NR	NR	NR
Mao et al. (2016)		NR	NR	NR	Caudate nucleus (volume) – ‘pain characteristics’	NR	NS (NR)
Reckziegel et al. (2016)	Knee				**Gamma-aminobutyric acid—cingulate**	– **0.76**	** < 0.001**
					Glu + glutamine	NR	NS (NR)
Ushio et al. (2020)	Knee	NR	NR	NR	**Left anterior insular cortex-right orbitofrontal cortex functional connectivity – VAS**	**0.49**	**0.03**
					Left anterior insular cortex-right orbitofrontal cortex functional connectivity – WOMAC pain	0.26	0.28
					**Right anterior insular cortex-right orbitofrontal cortex functional connectivity – VAS**	**0.46**	**0.049**
					Right anterior insular cortex-right orbitofrontal cortex functional connectivity – WOMAC pain	0.26	0.28
Weerasekera et al. (2021)	Knee	NR	NR	NR	**Myoinositol (creatine referenced) – WOMAC pain**	**0.37**	** < 0.05**
					**Myoinositol (water referenced) – WOMAC pain**	**0.52**	** < 0.01**
					N-acetylasparate (created or water referenced) – WOMAC pain	0.30	≥ 0.09
					Choline (created or water referenced) – WOMAC pain	≤ 0.30	≥ 0.09

*NR* not reported, *NS* not significant, *NRS* numeric rating scale, *WOMAC* Western Ontario and McMaster Universities Arthritis Index, *VAS* visual analogue scale.

Significant values are in bold.

### Study quality

Study quality scores are shown in Table 4. Scores ranged from low to very low, with the majority of studies (23 of 28) rated as very low.

**Table 4:** Study quality assessment according to the National Institute of Health Quality Assessment Tool.

	Item 1	Item 2	Item 3	Item 4	Item 5	Item 6	Item 7	Item 8	Item 9	Item 10	Item 11	Item 12	Item 14	Overall Quality
Alshuft et al. (2016)	✓	×	-	×	✓	×	×	✓	×	×	-	✓	×	Very low
Baliki et al. (2011)	✓	✓	-	×	×	×	×	✓	×	×	-	–	-	Very low
Baliki et al. (2014)	✓	✓	-	×	×	×	×	✓	×	×	-	–	✓	Very low
Barroso et al. (2020)	✓	✓	-	✓	×	×	×	×	×	×	-	–	-	Very low
Barroso et al. (2021)	✓	✓	-	✓	×	×	×	✓	×	×	-	–	✓	Very low
Cheng et al. (2022)	✓	✓	-	✓	×	×	×	×	✓	×	-	–	✓	Low
Cottam et al. (2016)	✓	✓	-	×	×	×	×	✓	×	×	-	–	✓	Very low
Cottam et al. (2018)	✓	×	-	×	×	×	×	✓	×	×	-	–	✓	Very low
El-Najjar et al. (2020)	✓	✓	-	-	×	×	×	✓	-	×	-	–	×	Very low
Gandola et al. (2017)	✓	×	-	-	×	×	×	✓	×	×	-	–	✓	Very low
Gwilym et al. (2010)	✓	✓	-	-	×	×	×	✓	✓	×	-	–	×	Very low
Hiramatsu et al. (2014)	✓	×	-	-	×	×	×	✓	✓	×	-	–	×	Very low
Howard et al. (2012)	✓	×	-	-	×	×	×	×	✓	×	-	–	✓	Very low
Iwabuchi et al. (2020)	✓	✓	✓	-	✓	×	×	✓	✓	×	-	–	-	Low
Kang et al. (2022)	✓	✓	-	✓	×	×	×	×	×	×	-	–	✓	Low
Lan et al. (2020)	✓	×	✓	×	×	×	×	✓	×	×	-	–	✓	Very low
Lewis et al. (2018)	✓	✓	-	✓	✓	×	×	✓	✓	×	-	–	✓	Very low
Liao et al. (2018)	✓	✓	-	-	×	×	×	✓	-	×	-	–	✓	Very low
Mao et al. (2016)	✓	✓	-	×	×	×	×	✓	×	×	-	–	✓	Very low
Mutso et al. (2012)	✓	✓	-	×	×	×	×	×	×	×	-	–	–-	Very low
Railton et al. (2022)	✓	✓	-	-	×	×	×	✓	×	×	-	–	✓	Low
Reckziegel et al. (2016)	✓	×	-	×	×	×	×	✓	×	×	-	–	-	Very low
Rodriguez-Raecke et al. (2013)	×	×	-	-	×	×	×	×	✓	×	-	–	×	Very low
Rodriguez-Raecke et al. (2009)	×	×	-	-	×	×	×	×	✓	×	-	–	×	Very low
Russell et al. (2018)	✓	✓	-	-	✓	×	×	×	✓	×	-	–	✓	Very low
Tetreault et al. (2018)	✓	✓	-	×	×	×	×	×	×	×	-	–	✓	Very low
Ushio et al. (2020)	✓	×	-	-	×	×	×	✓	✓	×	-	✓	✓	Very low
Weerasekera et al. (2021)	✓	✓	-	✓	×	×	×	✓	×	×	-	×	✓	Low

✓ represents yes, × represents no,—represents could not determine, – represents not reported, –- represents not applicable. Item 13 was not applicable for all studies.

## Discussion

The aims of this systematic review and meta-analysis were to (1) establish the evidence for alterations in structure and function of the brain in people with OA and (2) investigate the association between changes in brain structure and function and OA joints, pain severity, and duration. Our primary ALE meta-analysis did not show any differences in the brain structure or function between people with OA and healthy controls. Findings from our sensitivity analysis implicated the left post central gyrus in OA. Most studies evaluated knee OA, with only a few studies focusing on hip and hand OA. Findings for our exploratory ALE meta-analysis of studies that reported OA less than healthy controls contrasts converge with the narrative synthesis to suggest that the right anterior insula is the brain region that may be implicated in OA. People with OA may have less brain activity, connectivity and volume compared to healthy controls in this brain region. Indeed, the right anterior insula was implicated in knee OA and hip OA when compared separately to healthy controls. Notably, differences between hip OA compared to healthy controls were also observed in the medial prefrontal cortex. There was minimal evidence to suggest that pain intensity or pain duration associate with changes in brain structure and function. This systematic review was conducted in accordance with best practices of neuroimaging analysis^10,11^, yet the quality of studies informing the body of evidence was considered low. Thus, we have limited certainty in the robustness of our findings.

The impetus for this systematic review and ALE meta-analysis was the observation of inconsistent results in studies investigating brain structure and function in OA, and the subsequent difficulty in selecting a marker(s) of brain structure and function to understand response to treatments for OA. Indeed, pooling data from all available studies for analysis did not reveal significant differences between those with OA and healthy controls. Although this finding may indeed suggest no difference in brain or function, differences may be undetectable. Symptom heterogeneity along with the heterogeneity of techniques used to assess the brain are possible explanations for the overall absence of differences between OA and healthy controls in our primary analysis. To overcome the issue of various MRI approaches, we isolated the ALE meta-analysis to specific techniques (e.g. MRI structural). However, no differences were observed which perhaps stems from the remaining issue of heterogeneity among the participants. One approach may be to assess subgroups of OA based on symptoms, as it could be reasonably speculated that people with more intense pain and/or longer duration of symptoms may have more pronounced brain adaptations. However, the challenge of identifying homogenous subgroups of people with OA is highlighted by the general lack of association between brain measures and pain characteristics including intensity and duration (Table 3). The absence of association between potential markers of OA and clinical pain is an issue that extends beyond the brain imaging field (e.g. biomechanics^41^), and again questions our rudimentary tools to assess pain (e.g. VAS, NRS). Notably, patients with OA struggle to self-describe pain with just “intensity” and describe numerous characteristics that vary in duration, depth, type of occurrence, impact and rhythm^42^.

The insula was most consistently implicated in several studies comparing OA and healthy controls, and also in association with pain intensity in our narrative review. Moreover, the right anterior insula emerged as significantly different between OA and healthy controls when including only studies that report differences of OA less than healthy controls. Although confirmatory studies are needed, we speculate these findings collectively suggest the insular cortex, and particularly the right anterior aspect may be implicated in the pathophysiology of OA. The insular cortex plays a role in somatosensory and pain processing in the central nervous system^43^ and the anterior insula plays a role in emotion experience and subjective feeling associated with nociception^43^. Hence, the potentially lower right insular volume in OA compared to healthy controls might imply the dysfunction of the right insula in interoceptive awareness and emotionally relevant context for sensory experience that contributes to OA pain. The insula is connected to various other structures associated with pain processing including but not limited to the cingulate, para hippocampal, precuneus, amygdala, medial prefrontal cortex and occipital regions^44,45^, that were also identified as different to healthy controls, albeit less consistently. It remains unclear whether the potential alterations in the insula associated with OA drive adaptations to other structures and functions of the brain through its elaborate connectivity to many other structures.

We observed that knee and hip OA exploratory contrasts did not yield completely identical results. Specifically, hip OA was additionally associated with the cluster in medial prefrontal cortex, suggesting that there might be differences between OA types in the brain. Although people with hip and knee OA are often studied together^39,40^, there are differences between hip and knee OA^46^. For example, robust qualitative research (143 participants) suggests that people with hip OA often use more intense language to describe their pain compared to those with knee OA^47^. The affective and cognitive components of the pain sensation are processed in subregions of the medial prefrontal cortex, which may link to differences in pain experiences between hip and knee OA^48^. The differences in medial prefrontal cortex were informed by contrasts to healthy controls and due to limited number of studies available we were not able to conduct a direct comparison between hip and knee OA. Studies typically do not exclude participants if they have OA in joint beyond the joint of investigation. Hence, caution should be used interpreting these findings between potential differences in osteoarthritic joints and controls, as OA often affects more than one joint. Future studies should specifically study differences between the brain organisation of different OA sites.

### Limitations and future directions

Our findings should be interpreted with caution considering some key limitations. First, we used a meta-analytic algorithm to integrate existing data and delineate consistent association across studies. However, this analytic approach can only include results from experiments that reach significance. Although, this limitation biases the meta-analysis toward finding significant results it adds confidence in our null finding from our primary ALE meta-analysis as we did not observe an association even when null experiments were included. Second, several factors such as sex^49^ and medication may play a role in brain structure and function adaptation, specific to the insula^49^ in people with chronic pain. However, the insufficient number of eligible experiments limited our ability to robustly assess the influence of these factors. The diverse inclusion criteria relating to medication used across experiments precludes subgroup analysis focused on medication. Third, most studies excluded participants with depression and anxiety. This may limit the generalisability of findings given the prevalence of depression and anxiety is approximately 20% in people with knee OA^50^, and evidence on the neural correlates of pain and depression^51^. Finally, limiting our focus to cross-sectional studies to better understand alterations associated with OA neglects understanding of longitudinal changes or changes in response to treatments. For example, longitudinal studies might provide insight into neuroplastic features associated that complement understanding of neuroplastic adaptations in OA beyond the brain^52^. Despite the difficulties associated with assessing pain, future research is encouraged to consider subgroups potentially based on pain characteristics. In fibromyalgia, Liu et al.^53^ eloquently demonstrated the neuroplastic potential of the right anterior insular cortex when subgrouping patients by number of painful sites. More studies with sample sizes appropriately powered to detect potentially meaningful differences will reduce heterogeneity in estimates and increase confidence in the estimate ranges of possible differences for different measures of neurobiology associated with OA. This is a rapidly changing field of research, and inclusion of new experiments may change our findings.

## Conclusions

In summary, our pre-registered analysis did not find evidence of significant differences in OA neurobiology compared to healthy controls. However, findings from our exploratory quantitative analysis converge with our narrative synthesis to suggest that the right insula – namely interoceptive awareness and emotionally relevant context for sensory experience that contributes to OA pain may be implicated in knee and hip OA. Some limited evidence also potentially implicates the medial prefrontal cortex in hip OA. Despite the limitations associated with heterogeneity and study quality, these regions are potentially relevant to OA provide avenues for future research.

## Supplementary Information

Supplementary Information 1.

Supplementary Information 2.

## Data Availability

All datasets generated and analysed during the current study, such as specific coordinates for the ALE analysis, are available in Supplementary Appendix 2.
