# Supplementary material for: Neurobiology of osteoarthritis: a systematic review and activation likelihood estimation meta-analysis
Source: Sci Rep. 2023 Aug 1;13:12442. doi: 10.1038/s41598-023-39245-9 (PMC10394087; doi:10.1038/s41598-023-39245-9)
Supplement: Supplementary file 1 — Supplementary Information 1. [file 41598_2023_39245_MOESM1_ESM.pdf]

Medline 10/2/21

Database(s): **Ovid MEDLINE(R) and Epub Ahead of Print, In-Process & Other Non-Indexed Citations and Daily** 1946 to February 10, 2021
Search Strategy:

#	Searches	Results
1	brain*.mp. [mp=title, abstract, original title, name of substance word, subject heading word, floating sub-heading word, keyword heading word, organism supplementary concept word, protocol supplementary concept word, rare disease supplementary concept word, unique identifier, synonyms]	1479258
2	exp Brain/	1224876
3	neuroimag*.mp. [mp=title, abstract, original title, name of substance word, subject heading word, floating sub-heading word, keyword heading word, organism supplementary concept word, protocol supplementary concept word, rare disease supplementary concept word, unique identifier, synonyms]	65337
4	exp Neuroimaging/	175141
5	1 or 2 or 3 or 4	1973412
6	(osteoarthrit* or osteo arthritis or osteoarthos* or osteo arthros*).mp. [mp=title, abstract, original title, name of substance word, subject heading word, floating sub-heading word, keyword heading word, organism supplementary concept word, protocol supplementary concept word, rare disease supplementary concept word, unique identifier, synonyms]	94965
7	exp Osteoarthritis/	65146
8	6 or 7	94965
9	5 and 8	521
10	from 9 keep 1-517	517

Embase 10/2/21

Database: Embase Classic+Embase <1947 to 2021 February 10>
Search Strategy:
----
1 brain*.mp. (2373609)
2 exp brain/ (1575592)
3 neuroimag*.mp. [mp=title, abstract, heading word, drug trade name, original title, device manufacturer, drug manufacturer, device trade name, keyword, floating subheading word, candidate term word] (174842)
4 exp neuroimaging/ (144785)
5 1 or 2 or 3 or 4 (2827158)
6 (osteoarthrit* or osteo arthritis or osteoarthos* or osteo arthros*).mp. [mp=title, abstract, heading word, drug trade name, original title, device manufacturer, drug manufacturer, device trade name, keyword, floating subheading word, candidate term word] (159044)
7 exp osteoarthritis/ (144048)
8 6 or 7 (167702)
9 5 and 8 (2913)

****

Psycinfo

Database(s): **APA PsycInfo** 1806 to February Week 1 2021
Search Strategy:

#	Searches	Results
1	brain*.mp. [mp=title, abstract, heading word, table of contents, key concepts, original title, tests & measures, mesh]	405745
2	exp brain/	270607
3	neuroimag*.mp. [mp=title, abstract, heading word, table of contents, key concepts, original title, tests & measures, mesh]	36787
4	exp neuroimaging/	101042
5	1 or 2 or 3 or 4	530009
6	(osteoarthrit* or osteo arthrit* or osteoarthos* or osteo arthros*).mp. [mp=title, abstract, heading word, table of contents, key concepts, original title, tests & measures, mesh]	2252
7	5 and 6	86

(No appropriate headings in Psycinfo for Osteoarthritis)

Cinahl

S1	brain*	**Search modes** - Boolean/Phrase	**View Results** (197,570) **View Details** **Edit**
S2	(MH "Brain+")	**Search modes** - Boolean/Phrase	**View Results** (95,268) **View Details** **Edit**
S3	neuroimag*	**Search modes** - Boolean/Phrase	**View Results** (11,404) **View Details** **Edit**
S4	(MH "Neuroradiography+")	**Search modes** - Boolean/Phrase	**View Results** (12,510) **View Details** **Edit**
S5	(S1 OR S2 OR S3 OR S4)	**Search modes** - Boolean/Phrase	**View Results** (226,580) **View Details** **Edit**

S6	(osteoarthrit* or "osteo arthrit*" or osteoarthos* or "osteo arthros*")	**Search modes** - Boolean/Phrase	**View Results** (38,946)<br>**View Details**<br>**Edit**
S7	(MH "Osteoarthritis+")	**Search modes** - Boolean/Phrase	**View Results** (29,486)<br>**View Details**<br>**Edit**
S8	S6 OR S7	**Search modes** - Boolean/Phrase	**View Results** (39,244)<br>**View Details**<br>**Edit**
S9	S5 AND S8	**Search modes** - Boolean/Phrase	**View Results** (229)

Scopus

Search history — Combine queries... e.g. #1 AND NOT #3

6	(TITLE-ABS-KEY ((osteoarthrit* OR "osteo arthrit*" OR osteoarthos* OR "osteo arthros*"))) AND ((TITLE-ABS-KEY (brain*)) OR (TITLE-ABS-KEY (neuroimag*)))	1,567 document results
5	(TITLE-ABS-KEY (brain*)) OR (TITLE-ABS-KEY (neuroimag*))	2,286,922 document results
4	TITLE-ABS-KEY ((osteoarthrit* OR "osteo arthrit*" OR osteoarthos* OR "osteo arthros*"))	131,674 document results
2	TITLE-ABS-KEY (neuroimag*)	168,085 document results
1	TITLE-ABS-KEY (brain*)	2,248,450 document results

^ Top of page

Web of Science

# 5	612	#4 AND #3<br>*Indexes=SCI-EXPANDED, SSCI, A&HCI, CPCI-S, CPCI-SSH, BKCI-S, BKCI-SSH, ESCI, CCR-EXPANDED, IC Timespan=All years*
# 4	1,479,987	#2 OR #1<br>*Indexes=SCI-EXPANDED, SSCI, A&HCI, CPCI-S, CPCI-SSH, BKCI-S, BKCI-SSH, ESCI, CCR-EXPANDED, IC Timespan=All years*
# 3	100,904	**TOPIC:** ((osteoarthrit* or "osteo arthrit*" or osteoarthos* or "osteo arthros*"))<br>*Indexes=SCI-EXPANDED, SSCI, A&HCI, CPCI-S, CPCI-SSH, BKCI-S, BKCI-SSH, ESCI, CCR-EXPANDED, IC Timespan=All years*
# 2	59,669	**TOPIC:** (neuroimag*)<br>*Indexes=SCI-EXPANDED, SSCI, A&HCI, CPCI-S, CPCI-SSH, BKCI-S, BKCI-SSH, ESCI, CCR-EXPANDED, IC Timespan=All years*
# 1	1,457,583	**TOPIC:** (brain*)<br>*Indexes=SCI-EXPANDED, SSCI, A&HCI, CPCI-S, CPCI-SSH, BKCI-S, BKCI-SSH, ESCI, CCR-EXPANDED, IC Timespan=All years*

Updated results – Searches run 14/2/2022

All limited to published 2021-Current
Medline:

Database(s): **Ovid MEDLINE(R) and Epub Ahead of Print, In-Process, In-Data-Review & Other Non-Indexed Citations and Daily** 1946 to February 11, 2022
Search Strategy:

#	Searches	Results
1	brain*.mp. [mp=title, abstract, original title, name of substance word, subject heading word, floating sub-heading word, keyword heading word, organism supplementary concept word, protocol supplementary concept word, rare disease supplementary concept word, unique identifier, synonyms]	1552406
2	exp Brain/	1280360
3	neuroimag*.mp. [mp=title, abstract, original title, name of substance word, subject heading word, floating sub-heading word, keyword heading word, organism supplementary concept word, protocol supplementary concept word, rare disease supplementary concept word, unique identifier, synonyms]	72098
4	exp Neuroimaging/	186551
5	1 or 2 or 3 or 4	2063037
6	(osteoarthrit* or osteo arthritis or osteoarthos* or osteo arthros*).mp. [mp=title, abstract, original title, name of substance word, subject heading word, floating sub-heading word, keyword heading word, organism supplementary concept word, protocol supplementary concept word, rare disease supplementary concept word, unique identifier, synonyms]	102185
7	exp Osteoarthritis/	70703
8	6 or 7	102185
9	5 and 8	588
10	limit 9 to yr="2021 -Current"	76

Embase
Database(s): **Embase Classic+Embase** 1947 to 2022 February 11
Search Strategy:

#	Searches	Results
1	brain*.mp.	2491780
2	exp brain/	1633258
3	neuroimag*.mp. [mp=title, abstract, heading word, drug trade name, original title, device manufacturer, drug manufacturer, device trade name, keyword heading word, floating subheading word, candidate term word]	190449
4	exp neuroimaging/	158279
5	1 or 2 or 3 or 4	2962630
6	(osteoarthrit* or osteo arthritis or osteoarthos* or osteo arthros*).mp. [mp=title, abstract, heading word, drug trade name, original title, device manufacturer, drug manufacturer, device trade name, keyword heading word, floating subheading word, candidate term word]	170064

7	exp osteoarthritis/	153432
8	6 or 7	179058
9	5 and 8	3274
10	limit 9 to yr="2021 -Current"	345

Psycinfo

Database(s): **APA PsycInfo** 1806 to February Week 1 2022

Search Strategy:

#	Searches	Results
1	brain*.mp. [mp=title, abstract, heading word, table of contents, key concepts, original title, tests & measures, mesh word]	424849
2	exp brain/	282202
3	neuroimag*.mp. [mp=title, abstract, heading word, table of contents, key concepts, original title, tests & measures, mesh word]	40320
4	exp neuroimaging/	108808
5	1 or 2 or 3 or 4	553790
6	(osteoarthrit* or osteo arthrit* or osteoarthos* or osteo arthros).mp. [mp=title, abstract, heading word, table of contents, key concepts, original title, tests & measures, mesh word]	2388
7	5 and 6	99
8	limit 7 to yr="2021 -Current"	13

Update: 28/6/22

Medline – update search to limit to year 2022 to current

8	6 or 7	105060	Advanced	Display Results	More
9	5 and 8	614	Advanced	Display Results	More
10	limit 9 to yr="2022 -Current"	43	Advanced	Display Results	More

Embase – update search limit to year 2022 to current

8	6 or 7	184148	Advanced	Display Results	More
9	5 and 8	3433	Advanced	Display Results	More
10	limit 9 to yr="2022 -Current"	146	Advanced	Display Results	More

Psycinfo – update search to limit to year 2022 to current

☐	7	5 and 6	103	Advanced	Display Results | More ▾	
☐	8	limit 7 to yr="2022 -Current"	2	Advanced	Display Results | More ▾	

Scopus: Used filter to limit to 2022 = 82 results

[redacted]

☐	6	((TITLE-ABS-KEY (neuroimag*)) OR (TITLE-ABS-KEY (brain*))) AND (TITLE-ABS-KEY (osteoarthrit* OR ... Show more	82 results
☐	5	((TITLE-ABS-KEY (neuroimag*)) OR (TITLE-ABS-KEY (brain*))) AND (TITLE-ABS-KEY (osteoarthrit* OI... Show more	1,915 results
☐	4	(TITLE-ABS-KEY (neuroimag*)) OR (TITLE-ABS-KEY (brain*))	2,463,481 results
☐	3	TITLE-ABS-KEY (osteoarthrit* OR "osteo arthrit*" OR osteoarthros* OR "osteo arthros*")	147,617 results
☐	2	TITLE-ABS-KEY (neuroimag*)	188,579 results
☐	1	TITLE-ABS-KEY (brain*)	2,420,295 results

- ☐

  ((TITLE-ABS-KEY (neuroimag*)) OR (TITLE-ABS-KEY (brain*))) AND (TITLE-ABS-KEY (osteoarthrit* OR "osteo arthrit*" OR osteoarthros* OR "osteo arthros*")) AND (LIMIT-TO (PUBYEAR , 2022))

  Show more

  82 resultsSet AlertMore

- ☐ 5Edit

  ((TITLE-ABS-KEY (neuroimag*)) OR (TITLE-ABS-KEY (brain*))) AND (TITLE-ABS-KEY (osteoarthrit* OR "osteo arthrit*" OR osteoarthros* OR "osteo arthros*"))

  Show more

  1,915 resultsSet AlertMore

- ☐ 4Edit

(TITLE-ABS-KEY (neuroimag*)) OR (TITLE-ABS-KEY (brain*))

2,463,481 resultsSet AlertMore

- ☐ 3Edit

  TITLE-ABS-KEY (osteoarthrit* OR "osteo arthrit*" OR osteoarthros* OR "osteo arthros*")

  147,617 resultsSet AlertMore

- ☐ 2Edit

  TITLE-ABS-KEY (neuroimag*)

  188,579 resultsSet AlertMore

- ☐ 1Edit

  TITLE-ABS-KEY (brain*)

Web of science – Limit to 2022- 52 documents

Search	#4 AND #3 and 2022 (Publication Years)	Web of Science Core Collection	52
	2:37 PM	Show editions	

Updated Endnote library – 28/6/22

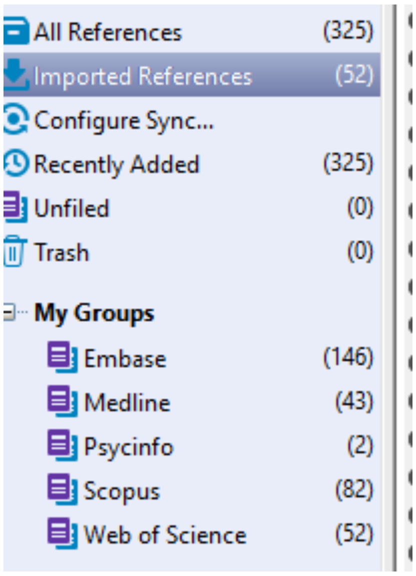

Updated search uploaded and sent to Michelle on 28/6 - TC
