# Supplementary material for: Neurobiology of osteoarthritis: a systematic review and activation likelihood estimation meta-analysis
Source: Sci Rep. 2023 Aug 1;13:12442. doi: 10.1038/s41598-023-39245-9 (PMC10394087; doi:10.1038/s41598-023-39245-9)
Supplement: Supplementary file 2 — Supplementary Information 2. [file 41598_2023_39245_MOESM2_ESM.pdf]

1. OA vs. healthy controls

// Reference=MNI
//Baliki, 2011
//Subjects=76
-42 -18 20
38 -14 20
30 -16 -12
-30 -18 -12
36 12 0
4 -24 52
-10 -24 48
0 -80 12

//Baliki, 2014
//Subjects=50
-4 58 2
2 36 22
-38 10 -12
-56 -36 26

//Barroso, 2020a
//Subjects=127
12 -58 10
30 24 50
-38 2 64

//Barroso, 2021
//Subjects=150
-2 35 31
52 -59 36
36 10 1
59 -17 29
37 1 -4
29 -17 71
-38 -27 69
-31 -10 -36
47 10 33
-23 -30 72
33 -12 -34

//Cottam, 2018
//Subjects=44
58 12 -2
-8 50 34
-10 -46 28
48 -68 30
-58 -58 30
-18 -80 2
-14 -52 52

//Gandola, 2017
//Subjects=70
-26 -24 70
-30 -28 66
46 -20 62

//Gwilym, 2010
//Subjects=32
-8 -20 14

8 -16 16
-34 14 6
-16 -6 -16
-30 -12 -46
28 -2 -50
26 -68 -62
-22 -58 -62
-4 -64 -60
-24 -78 -56
26 -28 -18
-46 20 -10
-44 -86 12
10 -96 20

//Hiramatsu, 2014
//Subjects=23
24 22 52
20 6 44
-16 44 42
-26 -86 46
30 -74 2
-14 -96 4
-38 -80 32

//Howard, 2012
//Subjects=33
-4 48 6
-36 -34 20
-36 -2 16
-10 -58 12
16 -58 24
-52 -16 22
-16 -38 78
-60 -30 48
40 -34 48
-24 -16 -26
-26 0 -24
-10 -26 0
-4 -30 -12
2 -31 -26
-6 46 -8
-10 -14 78
6 38 -10
-40 22 20
-40 4 24
-62 0 34
-48 -8 60
-56 8 6
38 -22 68
-4 -52 44
-16 -66 36
-10 -68 64
6 -78 44
6 -62 68
20 -62 38
-28 -78 48
-32 -56 56
-36 -36 44
-38 -60 38
-58 -52 50

46 -42 60
36 -62 42
30 -48 54
-56 -16 -2
-60 -54 18
-46 -64 18
-66 -10 -18
-62 -30 -22
-36 -50 -20
-50 -8 -28
-4 -88 18
16 -92 32
-6 -78 -2
-36 -90 12
-56 -64 -14
-44 -84 -18
-52 -52 -32
-32 -70 -40

//Iwabuchi, 2020
//Subjects=73
-14 -84 40
8 -88 -32
-42 -26 -22
54 -62 -12
-14 -42 -10
-40 -76 18
-26 -52 -56
-6 -50 -60
-4 -44 -62
-48 22 -24
4 -18 10
48 -6 -46
16 -68 -2
-26 -22 -20
-12 66 24
-44 -74 -16
-6 70 8
26 -10 -38
-16 -10 24
-58 -20 28
30 54 10
28 -38 -50
-44 -16 6
-2 42 10
0 30 -20
36 24 -8
42 -12 18
-48 -58 -50
52 -26 40
-32 52 6
0 -4 36
-34 30 -2
38 -32 42
54 10 32
42 -60 56
-24 -58 68
42 22 30
12 40 14
-28 -82 -44

56 10 12
-56 -10 -32
-58 -4 -2
12 10 62
-20 30 -22
40 10 48
-46 2 -12
-24 44 20
22 12 -16
-50 -52 28
10 10 58

//Lan, 2020
//Subjects=46
-11 -66 51
12 -76 47
-54 -60 30
53 -54 38
-12 63 12
13 59 20
-18 -51 -51
28 -53 -52
-26 0 -11
25 4 -16

//Lewis, 2018
//Subjects=47
40 39 72
51 66 31
41 66 32
59 59 25
32 65 28

//Liao, 2018
//Subjects=60
-6 16 -16
46 38 -12
10 -32 68

//Rodriguez-Raecke, 2013
//Subjects=40
-12 25 -16
54 22 5
26 -42 -27
48 47 -9
-14 68 2
-63 -29 -7
10 67 13
48 18 0
45 35 29
64 -5 4
31 -14 -1

//Rodriguez-Raecke, 2009
//Subjects=64
-4 39 3
28 1 -15
23 49 38
-11 -14 35
51 17 1

-41 26 4
2 -30 -48
-62 -17 0
12 23 24
-9 24 -22
6 62 -12
21 -6 59
27 9 -42
18 -43 -33
9 66 13
54 -32 53
-28 9 62

//Tetreault, 2018
//Subjects=59
-38 39 21
6 50 8
-2 -54 40
34 18 0
58 -30 24
-2 -6 32
-33 -33 69
-6 -35 3
-6 61 -5
-58 -62 8
61 -1 8
2 -38 -24
13 10 19

//Ushio, 2020
//Subjects=34
18 14 -30
14 20 -22
8 68 20

//Kang, 2022
//Subjects=74
-57 -18 -21
-63 -21 -3
-54 0 -36
24 48 39
-33 21 48
-6 57 36

2. OA > healthy controls

// Reference=MNI
//Barroso, 2021
//Subjects=150
29 -17 71
-38 -27 69
-31 -10 -36
47 10 33
-23 -30 72
33 -12 -34

//Cottam, 2018
//Subjects=44
-18 -80 2
-14 -52 52

//Gwilym, 2010
//Subjects=32
-34 14 6
-16 -6 -16
-30 -12 -46
28 -2 -50
26 -68 -62
-22 -58 -62
-4 -64 -60
-24 -78 -56
26 -28 -18
-46 20 -10
-44 -86 12
10 -96 20

//Hiramatsu, 2014
//Subjects=23
24 22 52
20 6 44
-16 44 42
-26 -86 46
30 -74 2
-14 -96 4
-38 -80 32

//Howard, 2012
//Subjects=33
-4 48 6
-36 -34 20
-36 -2 16
-10 -58 12
16 -58 24
-52 -16 22
-16 -38 78
-60 -30 48
40 -34 48
-24 -16 -26
-26 0 -24
-10 -26 0
-4 -30 -12
2 -31 -26
-6 46 -8
-10 -14 78

6 38 -10
-40 22 20
-40 4 24
-62 0 34
-48 -8 60
-56 8 6
38 -22 68
-4 -52 44
-16 -66 36
-10 -68 64
6 -78 44
6 -62 68
20 -62 38
-28 -78 48
-32 -56 56
-36 -36 44
-38 -60 38
-58 -52 50
46 -42 60
36 -62 42
30 -48 54
-56 -16 -2
-60 -54 18
-46 -64 18
-66 -10 -18
-62 -30 -22
-36 -50 -20
-50 -8 -28
-4 -88 18
16 -92 32
-6 -78 -2
-36 -90 12
-56 -64 -14
-44 -84 -18
-52 -52 -32
-32 -70 -40

//Iwabuchi, 2020
//Subjects=73
-14 -84 40
8 -88 -32
-42 -26 -22
54 -62 -12
-14 -42 -10
-40 -76 18
-26 -52 -56
-6 -50 -60
-4 -44 -62
-48 22 -24
4 -18 10
48 -6 -46
16 -68 -2
-26 -22 -20
-12 66 24
-44 -74 -16
-6 70 8
26 -10 -38
-16 -10 24

//Lan, 2020

//Subjects=46
-18 -51 -51
28 -53 -52
-26 0 -11
25 4 -16

//Rodriguez-Raecke, 2013
//Subjects=40
31 -14 -1

//Tetreault, 2018
//Subjects=59
-2 -6 32
-33 -33 69
-6 -35 3
2 -38 -24
13 10 19

//Ushio, 2020
//Subjects=34
18 14 -30
14 20 -22
8 68 20

3. OA < healthy controls

// Reference=MNI
//Baliki, 2011
//Subjects=76
-42 -18 20
38 -14 20
30 -16 -12
-30 -18 -12
36 12 0
4 -24 52
-10 -24 48
0 -80 12

//Baliki, 2014
//Subjects=50
-4 58 2
2 36 22
-38 10 -12
-56 -36 26

//Barroso, 2020a
//Subjects=127
12 -58 10
30 24 50
-38 2 64

//Barroso, 2021
//Subjects=150
-2 35 31
52 -59 36
36 10 1
59 -17 29
37 1 -4
29 -17 71
-38 -27 69
-31 -10 -36
47 10 33
-23 -30 72
33 -12 -34

//Cottam, 2018
//Subjects=44
58 12 -2
-8 50 34
-10 -46 28
48 -68 30
-58 -58 30

//Gandola, 2017
//Subjects=70
-26 -24 70
-30 -28 66
46 -20 62

//Gwilym, 2010
//Subjects=32
-8 -20 14
8 -16 16

//Iwabuchi, 2020
//Subjects=73
-58 -20 28
30 54 10
28 -38 -50
-44 -16 6
-2 42 10
0 30 -20
36 24 -8
42 -12 18
-48 -58 -50
52 -26 40
-32 52 6
0 -4 36
-34 30 -2
38 -32 42
54 10 32
42 -60 56
-24 -58 68
42 22 30
12 40 14
-28 -82 -44
56 10 12
-56 -10 -32
-58 -4 -2
12 10 62
-20 30 -22
40 10 48
-46 2 -12
-24 44 20
22 12 -16
-50 -52 28
10 10 58

//Lan, 2020
//Subjects=46
-11 -66 51
12 -76 47
-54 -60 30
53 -54 38
-12 63 12
13 59 20

//Lewis, 2018
//Subjects=47
40 39 72
51 66 31
41 66 32
59 59 25
32 65 28

//Liao, 2018
//Subjects=60
-6 16 -16
46 38 -12
10 -32 68

//Rodriguez-Raecke, 2013
//Subjects=40
-12 25 -16

54 22 5
26 -42 -27
48 47 -9
-14 68 2
-63 -29 -7
10 67 13
48 18 0
45 35 29
64 -5 4

//Rodriguez-Raecke, 2009
//Subjects=64
-4 39 3
28 1 -15
23 49 38
-11 -14 35
51 17 1
-41 26 4
2 -30 -48
-62 -17 0
12 23 24
-9 24 -22
6 62 -12
21 -6 59
27 9 -42
18 -43 -33
9 66 13
54 -32 53
-28 9 62

//Tetreault, 2018
//Subjects=59
-38 39 21
6 50 8
-2 -54 40
34 18 0
58 -30 24
-6 61 -5
-58 -62 8
61 -1 8

//Kang, 2022
//Subjects=74
-57 -18 -21
-63 -21 -3
-54 0 -36
24 48 39
-33 21 48
-6 57 36

4. OA vs. healthy controls as measured by resting-state MRI

// Reference=MNI
//Baliki, 2014
//Subjects=50
-4 58 2
2 36 22
-38 10 -12
-56 -36 26

//Barroso, 2021
//Subjects=150
-2 35 31
52 -59 36
36 10 1
59 -17 29
37 1 -4
29 -17 71
-38 -27 69
-31 -10 -36
47 10 33
-23 -30 72
33 -12 -34

//Cottam, 2018
//Subjects=44
58 12 -2
-8 50 34
-10 -46 28
48 -68 30
-58 -58 30
-18 -80 2
-14 -52 52

//Lan, 2020
//Subjects=46
-11 -66 51
12 -76 47
-54 -60 30
53 -54 38
-12 63 12
13 59 20
-18 -51 -51
28 -53 -52
-26 0 -11
25 4 -16

//Tetreault, 2018
//Subjects=59
-38 39 21
6 50 8
-2 -54 40
34 18 0
58 -30 24
-2 -6 32
-33 -33 69
-6 -35 3

//Ushio, 2020
//Subjects=34

18 14 -30
14 20 -22
8 68 20

//Kang, 2022
//Subjects=74
24 48 39
-33 21 48
-6 57 36

5. OA vs. healthy controls as measured by structural MRI

// Reference=MNI
//Baliki, 2011
//Subjects=76
-42 -18 20
38 -14 20
30 -16 -12
-30 -18 -12
36 12 0
4 -24 52
-10 -24 48
0 -80 12

//Barroso, 2020a: KOA vs HC
//Subjects=127
12 -58 10
30 24 50
-38 2 64

//Barroso, 2020a: HOA+KOA vs HC
//Subjects=151
8 -8 32
-28 -18 70
34 8 34

//Gwilym, 2010
//Subjects=32
-8 -20 14
8 -16 16
-34 14 6
-16 -6 -16
-30 -12 -46
28 -2 -50
26 -68 -62
-22 -58 -62
-4 -64 -60
-24 -78 -56
26 -28 -18
-46 20 -10
-44 -86 12
10 -96 20

//Lewis, 2018
//Subjects=47
40 39 72
51 66 31
41 66 32
59 59 25
32 65 28

//Liao, 2018
//Subjects=60
-6 16 -16
46 38 -12
10 -32 68

//Rodriguez-Raecke, 2013
//Subjects=40
-12 25 -16

54 22 5
26 -42 -27
48 47 -9
-14 68 2
-63 -29 -7
10 67 13
48 18 0
45 35 29
64 -5 4
31 -14 -1

//Rodriguez-Raecke, 2009
//Subjects=64
-4 39 3
28 1 -15
23 49 38
-11 -14 35
51 17 1
-41 26 4
2 -30 -48
-62 -17 0
12 23 24
-9 24 -22
6 62 -12
21 -6 59
27 9 -42
18 -43 -33
9 66 13
54 -32 53
-28 9 62

//Tetreault, 2018
//Subjects=59
-6 61 -5
-58 -62 8
61 -1 8
2 -38 -24
13 10 19

//Kang, 2022
//Subjects=74
-57 -18 -21
-63 -21 -3
-54 0 -36

6. Knee OA vs. healthy controls

// Reference=MNI
//Baliki, 2011
//Subjects=76
-42 -18 20
38 -14 20
30 -16 -12
-30 -18 -12
36 12 0
4 -24 52
-10 -24 48
0 -80 12

//Baliki, 2014
//Subjects=50
-4 58 2
2 36 22
-38 10 -12
-56 -36 26

//Barroso, 2020
//Subjects=127
12 -58 10
30 24 50
-38 2 64

//Barroso, 2021
//Subjects=150
-2 35 31
52 -59 36
36 10 1
59 -17 29
37 1 -4
29 -17 71
-38 -27 69
-31 -10 -36
47 10 33
-23 -30 72
33 -12 -34

//Cottam, 2018
//Subjects=44
58 12 -2
-8 50 34
-10 -46 28
48 -68 30
-58 -58 30
-18 -80 2
-14 -52 52

//Hiramatsu, 2014
//Subjects=23
24 22 52
20 6 44
-16 44 42
-26 -86 46
30 -74 2
-14 -96 4
-38 -80 32

//Iwabuchi, 2020
//Subjects=73
-14 -84 40
8 -88 -32
-42 -26 -22
54 -62 -12
-14 -42 -10
-40 -76 18
-26 -52 -56
-6 -50 -60
-4 -44 -62
-48 22 -24
4 -18 10
48 -6 -46
16 -68 -2
-26 -22 -20
-12 66 24
-44 -74 -16
-6 70 8
26 -10 -38
-16 -10 24
-58 -20 28
30 54 10
28 -38 -50
-44 -16 6
-2 42 10
0 30 -20
36 24 -8
42 -12 18
-48 -58 -50
52 -26 40
-32 52 6
0 -4 36
-34 30 -2
38 -32 42
54 10 32
42 -60 56
-24 -58 68
42 22 30
12 40 14
-28 -82 -44
56 10 12
-56 -10 -32
-58 -4 -2
12 10 62
-20 30 -22
40 10 48
-46 2 -12
-24 44 20
22 12 -16
-50 -52 28
10 10 58

//Lan, 2020
//Subjects=46
-11 -66 51
12 -76 47
-54 -60 30
53 -54 38

-12 63 12
13 59 20
-18 -51 -51
28 -53 -52
-26 0 -11
25 4 -16

//Lewis, 2018
//Subjects=47
40 39 72
51 66 31
41 66 32
59 59 25
32 65 28

//Liao, 2018
//Subjects=60
-6 16 -16
46 38 -12
10 -32 68

//Tetreault, 2018
//Subjects=59
-38 39 21
6 50 8
-2 -54 40
34 18 0
58 -30 24
-2 -6 32
-33 -33 69
-6 -35 3
-6 61 -5
-58 -62 8
61 -1 8
2 -38 -24
13 10 19

//Ushio, 2020
//Subjects=34
18 14 -30
14 20 -22
8 68 20

//Kang, 2022
//Subjects=74
-57 -18 -21
-63 -21 -3
-54 0 -36
24 48 39
-33 21 48
-6 57 36

7. Hip OA vs. healthy controls

// Reference=MNI
//Gwilym, 2010
//Subjects=32
-8 -20 14
8 -16 16
-34 14 6
-16 -6 -16
-30 -12 -46
28 -2 -50
26 -68 -62
-22 -58 -62
-4 -64 -60
-24 -78 -56
26 -28 -18
-46 20 -10
-44 -86 12
10 -96 20

//Rodriguez-Raecke, 2013
//Subjects=40
-12 25 -16
54 22 5
26 -42 -27
48 47 -9
-14 68 2
-63 -29 -7
10 67 13
48 18 0
45 35 29
64 -5 4
31 -14 -1

//Rodriguez-Raecke, 2009
//Subjects=64
-4 39 3
28 1 -15
23 49 38
-11 -14 35
51 17 1
-41 26 4
2 -30 -48
-62 -17 0
12 23 24
-9 24 -22
6 62 -12
21 -6 59
27 9 -42
18 -43 -33
9 66 13
54 -32 53
-28 9 62
